# Advancing a sociotechnical systems approach to workplace safety – developing the conceptual framework

**DOI:** 10.1080/00140139.2015.1015623

**Published:** 2015-04-02

**Authors:** Pascale Carayon, Peter Hancock, Nancy Leveson, Ian Noy, Laerte Sznelwar, Geert van Hootegem

**Affiliations:** ^a^ Department of Industrial and Systems Engineering, Center for Quality and Productivity Improvement, University of Wisconsin-Madison, Madison, USA; ^b^ Department of Psychology, University of Central Florida, Orlando, USA; ^c^ Department of Aeronautics and Astronautics, Massachusetts Institute of Technology, Cambridge, USA; ^d^ Liberty Mutual Research Institute for Safety, Hopkinton, USA; ^e^ Escola Politécnica da USP, University of São Paulo, São Paulo, Brazil; ^f^ Centre for Sociological Research, Katholieke Universiteit Leuven, Leuven, Belgium

**Keywords:** sociotechnical system, workplace safety, complexity, system levels, system interactions

## Abstract

Traditional efforts to deal with the enormous problem of workplace safety have proved insufficient, as they have tended to neglect the broader sociotechnical environment that surrounds workers. Here, we advocate a sociotechnical systems approach that describes the complex multi-level system factors that contribute to workplace safety. From the literature on sociotechnical systems, complex systems and safety, we develop a sociotechnical model of workplace safety with concentric layers of the work system, socio-organisational context and the external environment. The future challenges that are identified through the model are highlighted.

**Practitioner Summary:** Understanding the environmental, organisational and work system factors that contribute to workplace safety will help to develop more effective and integrated solutions to deal with persistent workplace safety problems. Solutions to improve workplace safety need to recognise the broad sociotechnical system and the respective interactions between the system elements and levels.

## 1. Introduction

Interest in the sociotechnical systems approach to workplace safety reflects a growing belief that many dimensions of safety are emergent properties of such systems. This evolution has resulted partly from the realisation that traditional strategies to address the enormous global burden of occupational injury may be reaching the limit of their utility. The particular limitations of ongoing efforts appear to be two-fold. First, risk management models that underlie scientific and professional approaches have only a limited ability to address latent and/or emergent risks and a restricted capacity to address the complexity of current and proposed work systems. Second, the focus on the individual worker misses many important phenomena when viewed from the broader sociotechnical systems perspective. We propose that meaningful advances in safety can be made if (1) there is a shift in the unit of analysis to the sociotechnical system level, which will thus incorporate human interdependencies relative to important social and technical elements, and (2) there is a meaningful expansion of the measurement and analysis methodologies so that assessment of systems dimensions, such as resilience and the adaptive role of workers in creating safety, can be more readily and more apparently revealed. In order to frame our argument, we first need to set it within the wider historical context of safety research.

Early medical interest in the adverse effects of work on health and safety is often traced to the seminal work by Ramazzini (1700), *De Morbis Artificum Diatriba* (*Diseases of Workers*). It would take nearly 200 more years to spark further significant scientific advances in workplace safety. In the USA, the catalyst was the Triangle Shirtwaist Factory fire of 25 March 1911 in New York City that claimed the lives of 146 workers (von Drehle 2004). This disaster impelled US state governments and employee groups to seek solutions to unsafe work conditions that had become widespread since the beginning of the Industrial Revolution. Such early efforts led to the creation of safety standards and legislation, and the establishment of professional organisations dedicated to improving worker safety. It also provided an impetus for safety research aimed at reducing work-related injuries and illnesses.

The global burden of work injury is enormous and persistent. The World Health Organization has estimated that in the year 2000 there were 2.0 million work-related deaths. In the USA, the Bureau of Labor Statistics estimates that nearly one million severe occupational injuries and nearly 5000 work-related deaths occur annually. The Liberty Mutual Workplace Safety Index (LMRIS Scientific Update 2012) has established that the direct costs of the most severe workplace injuries amount to approximately $50 billion annually. The search for ways to reduce this damage dictates that our focus shift from the individual to the broader context of work; namely the social, organisational and technical environment (Hendrick 1991; Kleiner 2006). Therefore, our paper proposes a sociotechnical systems approach to workplace safety, which is based on a larger tradition of sociotechnical systems research derived from a variety of domains and disciplines.

The benefit from the development of the study of sociotechnical systems is not confined to industrially strong societies. The same approaches are equally applicable in addressing the safety issues confronting industrially developing countries in order to avoid the experience of tragedy and workplace injury. The sociotechnical systems approach needs to be a targeted one both within and across such nations and indeed across the globe.

Workplace safety is not the absence of work-related injury. An enterprise can operate for a long period of time without mishap. However, that does not necessarily make it safe. Indeed, this record may simply be a stroke of good fortune, a statistical artefact, or more problematically it may relate to the way in which the organisation actually collects or categorises injury data that results in this apparently mishap-free situation. Similarly, the mere removal of specifically identified hazards does not necessarily make a system safer. Indeed, removal of one hazard might well create another, such as when collision risk is shuffled around the transportation system with changes to particular roadway configurations (Avgoustis, Rakha, and Van Aerde 2004). In this paper, workplace safety is defined as a system-level attribute of the degree of protection against harm afforded by that work system.

It is important to recognise that the academic distinction between system safety and workplace safety is actually not necessary. Most of the literature pertaining to system safety is concerned with the integrity of large-scale constructions such as power plants, spacecraft, mining operations and oil rigs. Catastrophic failures of any of these systems result not only in environmental and economic damage but also in physical harm to employees or bystanders as well as reduced public trust. Therefore, system safety is also concerned with safety of workers. In addition, the literature on system safety and workplace safety shares common concepts and methods. For instance, the concept of cumulative and latent failures or stressors is common to both system safety and workplace safety; this concept highlights the need to look at events that unfold over time that can produce ill health or accidents.

There are diverse areas, traditions and concepts within the overall field of human factors and ergonomics (HFE) that bear on the question of designing, planning and managing human interactions within complex work systems. These include, but are not limited to, macro-ergonomics, cognitive systems engineering, sociotechnical systems theory, anthropotechnology, psychodynamics of work, human-systems integration, organisational culture and safety climate, and resilience engineering. There are other traditions beyond HFE dealing with similar concerns within the management sciences, where the focus tends to be on organisational psychology, employee stress and behaviour. Many disparate scientific communities thus exist that deal with complex sociotechnical systems, and we need to create more opportunities for scientific contact and exchange between them. In the next section, we review and organise the various concepts, definitions and frameworks that relate to sociotechnical system safety, and describe their relevance to workplace safety.

## 2. Evolution towards a sociotechnical systems approach for workplace safety

### 2.1. Major limitations in current approaches to workplace safety

We describe two fundamental problems with the current research paradigm in workplace safety: (1) narrow identification of an injury event as a local failure in a system and (2) limited focus on exposure of the individual worker to workplace hazards.

The first concern with current research in workplace safety is about the conceptualisation of an injury event as a failure that can be localised within the system. Workplace safety science and practice have evolved to encompass two distinct levels that may be in effect at any particular organisation. The first level can be characterised as *reactive* and is based on the widely accepted hierarchy of controls. Hazards that are observed to cause injuries are dealt with in two steps: elimination if possible, and management of residual risk through administrative and/or engineering controls if not. The second level, characterised as preventive (or *proactive*) measures, recognises the value of anticipation over reactive approaches. In contrast to instituting measures in response to injury experience, the preventive approach integrates health and safety goals into an organisation's overall human resource and risk management strategy. However, this level, while embracing the proactive perspective, is also still fundamentally static in nature in the sense that it assesses present risks and develops interventions to manage those risks. The existing risk management paradigm, thus, effectively regards an injury event as a failure within the system (or a chain or confluence of failures of system components). Any solution involves iterating through the series of the following steps: identify priority hazards, isolate the causes, determine the mechanisms and develop countermeasures to protect workers from these hazards. If the hazard arises due to potential deficiencies in the interconnectedness of system components, it may well go undetected by current risk assessment approaches. We suggest that this paradigm, therefore, has limited ability to address emergent risks implicit in the whole system rather than any singular component.

A second problem, related to the first, is the traditional preoccupation with the exposure of the individual worker to workplace hazards. Much of the current literature in workplace safety focuses on identifying and mitigating factors that contribute to cumulative or traumatic injuries sustained by a particular worker performing a particular task. We recognise that the understanding of injury pathways, dose–response relationships and effectiveness of interventions to control such exposures has led to manifest reductions in injury frequency and disability. For example, between 1998 and 2006, occupational fatalities declined by nearly 20% in the 27 countries of the European Union (Eurostat 2013). Similarly, serious work-related injuries in the USA dropped by 32% over the same period (Bureau of Labor Statistics 2013). Despite these declining fatality and injury rates, however, the absolute magnitude of the occupational injury problem remains persistently and frustratingly high. There is a compelling reason to go beyond the individual and consider systemic hazards that arise from sociotechnical system attributes or functions that are organisational in nature.

### 2.2. Brief historical review of sociotechnical systems theory

Sociotechnical Systems (STS) theory was initially developed by members of the Tavistock Institute in London, with the primary objective to improve the overall quality of working life (for a review, see Mumford [2006]). A sociotechnical system is the synergistic combination of humans, machines, environments, work activities and organisational structures and processes that comprise a given enterprise. This conceptualisation primarily embraces complex systems in which many humans collaborate towards a common goal. A sociotechnical system has two inter-related sub-systems (Mumford 2006):

- the technology sub-system includes not only equipment, machines, tools and technology but also the work organisation;
- the social sub-system includes individuals and teams, and needs for coordination, control and boundary management.

The goal of STS is a comprehension and accounting for the ‘joint optimisation of the social and technical systems’, i.e. the different sub-systems or different system components. Joint optimisation involves interactions among system components, and between the system and its external environment (Hendrick and Kleiner 2001; Hancock 2009). Workers adapt to the sociotechnical system, but, in their turn, also serve to adapt the sociotechnical system itself. Such symbiotic interactions between workers and the rest of the sociotechnical system are further discussed below.

Interactions are key in STS theory. It is important to look at interactions between the sociotechnical system and the external environment it is situated in; this is in line with the concept of ‘open system’ (von Bertalanffy 1950; Katz and Kahn 1966). Systems Theory was first formulated in the 1930s and 1940s as a response to the limitations of the classic analysis technique of *analytic reduction* or the process of dividing a system into separate elements for analysis purpose. The genesis of Systems Theory was associated with efforts to cope with the increasingly complex systems then starting to develop. Weiner (1965) applied this approach to control and communications engineering, while von Bertalanffy (1968) developed similar ideas for biological systems. In traditional scientific and engineering methods, systems are divided into distinct parts that can then be examined separately in order to eventually seek comprehension of total system behaviour. Components of systems are decomposed into separate physical elements, while system behaviour is decomposed into discrete events. This decomposition assumes that the separation is feasible in both principle and approach, such that each component or sub-system operates independently and analytical results are not distorted when the components are considered independently. This assumption fails spectacularly for complex sociotechnical systems, where the interactions among components and events can be indirect and exhibit various forms that lead to the systems theory concept of *emergent* *properties.* These properties arise only when components interact and are not exhibited within the behaviour of individual components. We suggest that safety is one such emergent property.

Various conceptual approaches to sociotechnical systems differ along several dimensions. These include problem definition, view of the role of the human, theoretical precepts, scientific paradigms and methodologies (see Table 1). These ‘traditions’ intersect to an extent, but they also retain perspectives that are uniquely characteristic of their distinctive view of the system. A comprehensive review of these various approaches serves to generate valuable insight that can lead to innovative approaches to improving safety. One present aspiration is that it may lead to developing a comprehensive unified model of the human in sociotechnical systems that could stimulate new directions for scientific research. Our review here builds on and extends the review of sociotechnical systems approaches by Carayon (2006) and presents specific implications of the different sociotechnical systems approaches for workplace safety.

**Table 1:** STS approaches and implications for workplace safety (adapted from Carayon 2006).

Name of the approach/model (authors)	Purpose	Main elements or characteristics	View of the role of the human	Theoretical background/basis	Implications for workplace safety
STS theory (Trist and Bamforth 1951; Trist 1981; Mumford 2006)	Initially developed to improve quality of working life; further developments in design of information systems	Social sub-system	Human values; human needs	Open systems theory	Joint optimisation of technical and social sub-systems
		Technical sub-system		Psychology	
		Environment			
Wilson's (2000) Interacting Systems Model for Ergonomics	Systems approach to HFE	Person interacts with: tasks, hardware and software, other people, remote agents, structure-policy-roles, supply chain, environment, society-finance-politics	Individual at centre of system	HFE	Focus on system interactions
Model of Work System (Smith and Sainfort 1989; Carayon and Smith 2000; Carayon 2009)	Systemic approach to worker well-being and safety	Five elements of the work system: individual, task, tools and technologies, physical environment, organisation	Individual at centre of work system	Job design	Work system needs to be balanced to enhance worker well-being, health and safety
				Job stress	
				HFE	
Hendrick and Kleiner's macro-ergonomics approach (Hendrick and Kleiner 2001; Kleiner 2008)	Macro-ergonomics approach to work design	Personnel sub-system	Individual at centre of system	HFE	Different system levels
		Technological sub-system		STS theory	Joint optimisation of sociotechnical system
		Internal environment			
		External environment			
		Task and organisational design			
SHELL (Software-Hardware-Environment-Liveware) model (Rizzo et al. 2000)	Proactive approach to safety	Software: practices, procedures, regulations and formal/informal rules	System should support operator's work	HFE	Various system elements represent resources
		Hardware: physical elements of the sociotechnical system		Safety	Limited or inadequate resources affect safety
		Political, economic, social and legal environment in which the system functions			
		Liveware: workers and other people the workers interact with			
Moray (2000)	Systems approach to HFE	Individual behaviour, physical devices and physical ergonomics at the centre of the system	Individual at centre of system	HFE	Different system levels
		Other layers include team and group behaviour, organisational and management behaviour, legal and regulatory rules, and societal and cultural pressures			
Rasmussen (1997)	Systems approach to safety	System levels:	Adaptive role of workers	Control theory	Different system levels, and need for alignment between system levels
		Work		Safety	
		Staff			
		Management			
		Company			
		Regulators and associations			
		Government			
Human-Systems Integration (Booher 2003)	User-centred approach to complex systems design and deployment	HFE	Central focus of systems design and deployment; human-system performance is ultimate design criterion	HFE	Emphasis on human-system performance as central design criterion encompasses technical and organisational system design
		Safety		Systems engineering	
		Manpower		Training system design	
		Personnel		System safety	
		Survivability			
		Training			
		Health hazards			
Activity-related ergonomics or ergonomic work analysis (EWA) (Montmollin 1981, 1988; Wisner 1995; Daniellou 2004, 2006)	To transform work situations based on worker activities, and to promote health, quality and productivity	Integrated approach relating companies' strategies and actual working conditions	Human capacities and limits	Human physiology	Transformation of sociotechnical systems
		Discrepancy between (formal) tasks and (actual) activities	Deal with real work to create new ways to work and solve problems	Cognitive psychology	Design of safer systems
		Related to social movements and technological changes in order to adapt work to human characteristics		Anthropology	
Anthropotechnology (Wisner 1991)	To understand conditions and consequences related to companies implanted in industrially developing countries – technology transfer	Analysis at different levels relating the activities performed by workers (micro) to work organisation, industrial development and local culture (macro)	Dealing with adversity	Anthropology	To adapt technical systems, work organisation to actual conditions and afford different solutions for different situations
			Developing production systems in different social, technical, geographical, historical and cultural situations	Situated cognition	
Psychodynamics of work (Dejours 1980, 2009)	Relationship between work organisation and mental health	Worker engagement in order to achieve good results	Actions developing facing reality in order to guarantee production and respect of professional rules and traditions	Psychoanalysis	Design different work systems and organisation
	Goal is to improve conditions to improve the relation between workers and their work	Psychic defenses against suffering	Relationship between work organisation and mental health	HFE	Transform criteria to analyse worker performance
		Understanding worker behaviour in risky and hazardous conditions		Critical philosophy	
STAMP (Leveson 2012)	A new accident causality model to provide the basis for accident prevention, hazard analysis, design for safety, safer operations, and accident/incident investigations	Hierarchical safety control structure including engineering development, manufacturing, operations, and external stakeholders (e.g. the public). Integrates system and workplace safety.	Humans are components of the safety control structure, including legislators, regulators, managers, designers, operators, assemblers	Systems and control theory	The safety control structure, the physical design and the environment must be considered in designing work and investigating accidents

As highlighted in Table 1, the STS tradition considers the person as the centre of the system. The different approaches highlight the need to consider the physical, cognitive and psychosocial abilities and characteristics of individuals in the design of sociotechnical systems. The design of sociotechnical systems should aim to enhance quality of working life; this is another common element across the multiple STS approaches. For instance, Cherns (1976, 1987) described ‘high-quality work’ as jobs that (1) are reasonably physically and mentally demanding, (2) provide learning opportunities and allow workers to make work-related decisions in a supportive environment and (3) provide opportunities for a desirable job future. The goal of STS design is to design jobs with characteristics that promote workplace safety and health, such as those described by Cherns. Another common theme of STS is worker participation and involvement in system design; this is, for instance, in line with participatory ergonomics, a macro-ergonomic method (Noro and Imada 1991; Wilson and Haines 1997). Other STS approaches described in Table 1 (e.g. Wilson's interacting systems model for ergonomics and the model of work system by Smith and Carayon) emphasize the need for an active role of workers in sociotechnical system design. When workers participate in system design and implementation, numerous benefits occur, such as improved satisfaction and acceptance of change (Korunka, Weiss, and Karetta 1993), and improved integration of the technology into the work process (Carayon and Karsh 2000). Workers, therefore, play a central role in STS conceptualisation and a key role in sociotechnical systems implementation, in particular in ‘producing’ emergent workplace safety. Several STS approaches (e.g. ergonomics work analysis) also emphasize the distinction between what is conceived to and designed to happen, as opposed to what actually happens; this key issue is discussed in the next section.

## 3. The role of workers in workplace safety

### 3.1. Prescribed versus actual work: implications for emerging workplace safety

Approaches based on Scientific Management proposed by Taylor, and later developed by Ford, sought to prescribe work in the greatest possible level of detail in order to increase system predictability for production planning and control. As a result, it was critical to closely control, supervise and monitor work in production systems. In contrast, the STS perspective proposed by the Tavistock Institute came from a completely different epistemological perspective and, indeed, fundamental philosophical foundation. The original STS theory proposed to reduce the prescription of work to improve worker field of action (i.e. job control or autonomy) and to develop work systems based on semi-autonomous groups (Trist and Bamforth 1951; Trist 1981). According to the STS theory, it is not possible to organise and obtain results based only on deterministic rules. Actual work can be, and is, different from that which can be predicted, anticipated or prescribed. As rationality is limited, we are not able to anticipate every situation anyway. STS design principles of work systems should, therefore, support worker involvement and autonomy, which then enable workers to adapt to likely disturbances. Thus, we need different approaches to achieve performance goals; this leads to the present exploration of STS in the context of workplace safety.

Work activity analyses proposed by researchers in Belgium and France (e.g. Leplat 1989) have provided interesting results on the conflict between these two viewpoints, i.e. one proposing to maximise prescription versus the other that proposes autonomy and increased discretion for action. The discrepancy between what is prescribed and what is actually done is always present (Leplat 1989). Because of inherent uncertainty in any work system, it is not possible to reduce worker activities directly to the prescribed task, especially in information-based or cognitive work. Each task results from what was possible to design in a work system, including machines, tools, rules, goals, work division, time scheduling, work pace, quality goals and orders. Activity is related to how people actually behave in the scenario resulting from all these different components. Activity should be considered as the way the worker employs his/her physical, cognitive and psychosocial abilities to achieve performance goals (Dejours 2009).

In contrast to Scientific Management (Taylor 1911), the sociotechnical systems perspective and the Francophone ergonomics tradition assert that it is not possible to anticipate, control or find the appropriate person for every job. It is not feasible to understand the full range of worker behaviours without knowing their individual goals and needs. Sociotechnical systems should be considered more in terms of specific scenarios where people work and emergent phenomena (e.g. safety) may be anticipated. For instance, technologies often degrade after repeated use; therefore, it is necessary for organisations to provide preventive maintenance. However, maintenance is rarely instantaneously provided and so technologies are never in exactly the same condition as they were in the first use or, indeed, from week to week. This example demonstrates the need to incorporate the dynamic temporal aspects of sociotechnical systems into any model.

In order for us to make progress with this new direction, we must address the following question: Is it possible to design a safe system, and a system putatively immune to worker ‘errors’? Given the previous discussion asserting that it is not possible to anticipate all circumstances, in particular emerging situations, such design aspirations may be misguided. Systems are not only what was designed and intended in their initial conception but also influenced by many ongoing effects such as managerial decisions. Safety also depends on how people act to avoid accidents; this is the focus of resilience engineering (Hollnagel 2006). The main question should not be why accidents occur and whether people committed ‘errors’, but what people do in order to prevent and avoid accidents. Therefore, our focus is more on the dynamic ‘navigation’ of the system in the face of changing challenges and not on momentary events, which are minimally emblematic of change. Risks and hazards, therefore, always exist in operations and maintenance activities; some are known, others are not. The concept of human error alone is, therefore, insufficient in analysing accidents; factors contributing to accidents and injuries go beyond what happened at the time of the accident alone; they include factors in the large sociotechnical system (Reason 1997).

Workers are mostly engaged in their work; this explains why performance goals are frequently achieved. Workers often act with some degree of enthusiasm and are committed to their work. They may deviate from what was prescribed (Leplat 1989); this reflect workers' ability to strategise and deal with performance challenges (Dejours 2009). To avoid accidents and injuries, it is important to design and manage work systems that avoid reliance on a strict regimen of prescription.

### 3.2. Safety versus other goals: a sociotechnical viewpoint

Understanding the role of the worker in a sociotechnical system must include consideration of organisational and psychosocial factors in the workplace to complement our traditional physical and cognitive HFE approaches (Smith and Sainfort 1989; Carayon 2009). In the context of workplace safety, we, therefore, need to understand the potential conflicts of safety with other organisational goals and the role of safety climate and culture.

Frequently, what is espoused by senior management in terms of safety priorities does not reflect how safety-productivity trade-off decisions are actually made in the normal course of operations. Indeed, in profit-driven circumstances, conflicts between production and safety exist, and the question is whether STS can address and reconcile this eventual dissonance. In practice, workers are highly sensitive to real production priorities. In the normal course of work, an individual worker often encounters situations in which there is an inevitable conflict among strategic goals, operational demands (safety vs. speed), and contradictions between espoused and enacted priorities. How these conflicts are resolved (e.g. instruction of supervisors, discussion with peers) then influences the worker's perceptions of the true priority of safety in the organisation.

Woods (2006) has compared and contrasted the acute (short-term production goals) and the chronic (long-term) goals of an organisation. Safety is a chronic organisational goal that emerges from system interactions over a long period of time. Short- and long-term organisational goals are frequently in conflict. Therefore, it is necessary to clearly understand how to balance the trade-offs between the various goals (Woods 2006; Carayon 2009). Acute (short-term) goals naturally tend to bear greater momentary weight than chronic goals. So, Woods (2006) has suggested that safety should be put first as an organisational goal. These aspirations work well in a burgeoning economy where the value of safety can be emphasized as part of a growing organisation. However, in diminishing circumstances in which cuts, lay-offs, reductions and downsising occur, the emphasis on safety naturally erodes. Modern sociotechnical systems are transformative production systems at their very heart. Therefore, the great advantage of the STS approach is to recognise this necessary trade-off and to study its impact in a scientific manner. Further, a theoretical and empirical issue is whether these system goals must inevitably come into conflict or whether STS can distill an integrative path to the future.

Discussion of the organisational aspects of STS and workplace safety would not be complete without commenting on the growing literature on safety culture and safety climate. This literature seeks to understand how organisational values cascade down to individual workers and what impact they have on safety outcomes (Smith et al. 1978; Flin et al. 2000; DeJoy et al. 2004; Zohar 2010; Murphy, Robertson, and Carayon 2014). Safety climate is defined as ‘workers' shared perception of an organization's policies, procedures, and practices as they relate to safety priorities within the organization’. (Huang, Chen, and Grosch 2010, 1421). A number of studies have established that safety climate is a significant leading indicator of safety performance (Zohar 2010). Workers adapt their behaviours to meet corporate expectations and this, in turn, influences their risk of injury. However, it remains important to distinguish between climate and culture. The latter refers to a core set of values and beliefs shared by most members in an organisation; the former is often conceptualised as a measureable aspect of those core values, typically assessed through employee surveys (Guldenmund 2000, 2010).

As described above in the review of various STS approaches (see Table 1), an element of the STS process is to allow workers and teams to provide input and get involved in decisions regarding the design and implementation of sociotechnical systems. This allows workers and their colleagues to discuss various goals, possibly without compromising safety and health. This is a type of ‘space of deliberation’ (Dejours 2009) where conflicts are clearly laid out and different groups can achieve a compromise and balance production needs. The STS approach may actually be a unique path to discuss, integrate and balance various organisational goals, including workplace safety (Carayon and Smith 2000). Further research is necessary to describe the STS design characteristics and processes that can allow this type of balancing to occur.

## 4. Developing a sociotechnical systems approach to workplace safety

Complex work systems can be characterised by high uncertainty, multiple interacting elements and dynamic change (Vicente 1999; Carayon 2006). Human performance within these complex work systems can only be properly understood as human interactions within the broader context of work. These include both tangible (technology, physical environment) and intangible (psychosocial culture, work practices and procedures) elements and environments. It is also important to consider how work systems evolve over time and space and, thus, their sociotechnical attributes are constantly in flux. The time dimension can never be ignored. Systems approaches need to give sufficient consideration to system interfaces, system interactions and cross-level interactions (Waterson 2009; Karsh, Waterson, and Holden 2014). As we have established, a true sociotechnical systems approach to workplace safety needs to focus on safety as an emergent property and should, therefore, give pre-eminence to system interactions. In this section, we first review the Systems Theoretic Accident Modeling and Processes (STAMP) model that outlines levels of sociotechnical systems. We then propose a sociotechnical systems model for workplace safety that describes how safety in the local work context relates to the socio-organisational context and the external environment.

### 4.1. Levels of sociotechnical systems and implications for workplace safety

Rasmussen (1997) was the first to start moving away from the standard engineering *chain-of-events* model of accidents, which is based on reliability theory and component reliability. In this traditional model, system component failures lead to other component failures and eventually to an incident or loss event. Most of the activities in engineering to prevent accidents are based on this chain-of-events model and on reliability theory, which focuses on the reliability of each system component. Rasmussen proposed an alternative model, based on event chains, but that also includes the influence of social systems on the event chain. Although others had written papers about the influence of social and managerial factors on accidents, Rasmussen was the first to provide a fundamental engineering model that combined both the social and technical systems in an explanatory way.

The other important contribution made by Rasmussen (1997) was to create a theory of migration towards accidents. Most engineers think of engineered systems as static but Rasmussen emphasized the dynamic part of systems and accidents, and described how systems migrate towards states of high risk under competitive and economic pressures. In general, he emphasized that an accident is a complex process and not just a sequential chain of directly related events.

Rasmussen's (1997) model adds hierarchical control levels above the accident event chain to help explain why the events occurred and the influence of the social and managerial factors on the events. Svedung created an accident modelling procedure called AcciMaps, based on Rasmussen's model (see Svedung and Rasmussen 2002). AcciMaps appear similar to the model of accident causation proposed by Johnson (1980). Johnson also added hierarchical layers above the basic event chain where causes arise from contributory factors, which in turn arise from systemic factors. Both AcciMaps and the Johnson model attempted to expand fault trees (which are themselves based on chains of failure events) to include more types of information in the tree. Johnson called these Risk Trees, while Svedung called them AcciMaps; but both are enhancements of basic fault trees. Leveson (2012) started from Rasmussen's model but extended it further from traditional approaches and created a new model of accident causation called STAMP. STAMP is based on systems theory rather than reliability theory. The critical change is to consider accidents as a control problem rather than a component failure problem.

In systems theory, complex systems can be modelled as a *hierarchy* of levels of organisation, each more complex than its preceding level. Organisational levels are characterised by emergent properties that do not exist at lower levels; they are opaque to the language appropriate to those levels. The operation of processes at lower levels of the hierarchy results in a greater level of complexity at the higher levels. Hierarchy theory explains the relationships between differing levels, which can be thought of as control loops. Emergent properties (including safety) associated with a particular level of the hierarchy are related to *constraints upon the degree of freedom* of the interacting components at the level below. To effect control on emergent properties, the higher levels impose constraints on the behaviour of the level below.

**Figure 1:**
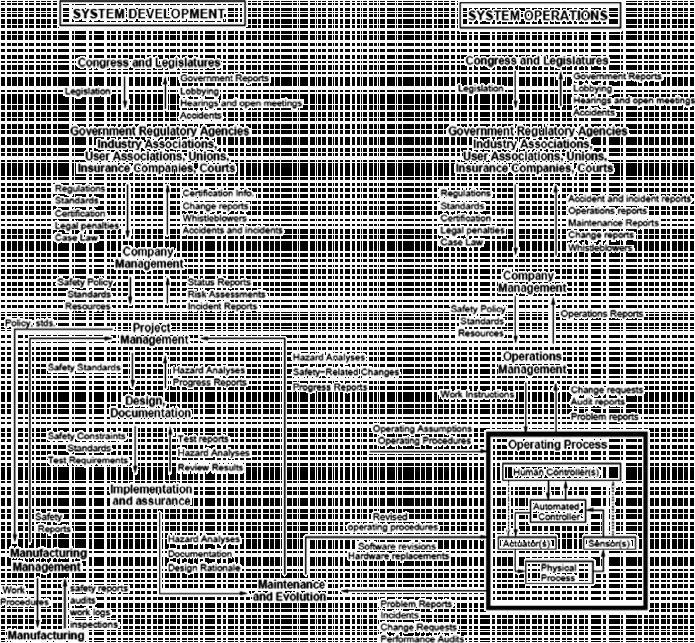
A general model of a sociotechnical safety control structure in STAMP (Leveson 2012).

Using systems theory as the basis for STAMP, safety is treated as an emergent property that results from the interactions among components in the system and controlled through the hierarchical safety control structure (Leveson 2012). An example of hierarchical safety control structure from STAMP is shown in Figure 1. It is similar in certain respects to Rasmussen's hierarchical model, but Rasmussen focused on system operations with little emphasis on system design (aside from it providing inputs to operations). Figure 1 shows the system design process on the left side and the system operations on the right side. Each component in the hierarchical safety control structure has responsibilities for enforcing safety constraints on the components it controls. Together, these responsibilities should result in enforcement of the overall system safety constraints necessary to address risk. When designing a new system or analysing an existing system using STAMP as the foundation, required safety constraints are first identified at the system level and then a top-down, iterative process is used to identify the required behavioural safety constraints that must be imposed at each of the lower level components. Controls are then designed to maintain the safety constraints. The entire safety control structure must be carefully designed and evaluated to ensure that the designed controls are adequate to maintain the constraints on behaviour necessary to control risk. Such safety control structures are not static, but change over time.

Unsafe system behaviour stems from the behavioural safety constraints not being met (Leveson 2012). The process that may lead to failing control (or an accident) can be very complex and may involve indirect, non-linear and feedback relationships among the events and the system components and not simply componential failures. We emphasize changing the focus of safety from ‘prevent failures’ to ‘enforce the safety constraints’. Enforcing these constraints may well involve preventing component failures, but it may also include enforcing constraints on the behaviour of the system and system components even when these have not ‘failed’. That is, it includes a larger set of causes of accidents, including sociotechnical factors, than are traditionally considered in purely mechanical systems. Standard control loops exist between system components.

In basic systems and control theory, an effective controller must contain a model of the system it is controlling, i.e. the *Process Model*. For human controllers, this model is usually called a *mental model*. Accidents in complex systems, particularly those with software or human controllers, often result from inconsistencies between the controller's process model and the actual process state. For example, the pilot fails to recognise that the aircraft is in a stall and applies incorrect control actions or the pilot thinks they have identified an aircraft as hostile and inappropriately shoots a missile at a friendly aircraft. A workplace safety example is when a worker thinks the catwalk will hold his or her weight but it will not.

Often, these models of the controlled system become inconsistent with the true state of the system due to missing or inadequate feedback channels. Part of the challenge in designing effective safety controls is providing feedback and inputs necessary to keep the controller's model consistent with the actual state of the process. Here, the concept of process models provides a better explanation for the human contribution to accidents than a simple ‘operator failure’. Operators (and administrators) do not fail in the ways that hardware fails; their contribution to accidents is much more complex. The system operation and working conditions must be controlled through physical, procedural and social (cultural) controls where the system component may be physical or human. STAMP provides a conceptual framework for designing systems to reduce human error that does not treat human error like machine failure.

In summary, while Rasmussen took an important step away from the traditional, overly simplistic, engineering models of accident causation, Leveson extended Rasmussen's conceptualisation to include an adaptive feedback function that fails to maintain safety as performance changes over time (Leveson 2012). The accident or loss itself results not simply from component failure or human error (which are symptoms rather than root causes) but from the inadequate control (i.e. enforcement) of safety-related constraints on the development, design, construction and operation of the entire sociotechnical system.

### 4.2. Development of a sociotechnical systems model for workplace safety

**Figure 2:**
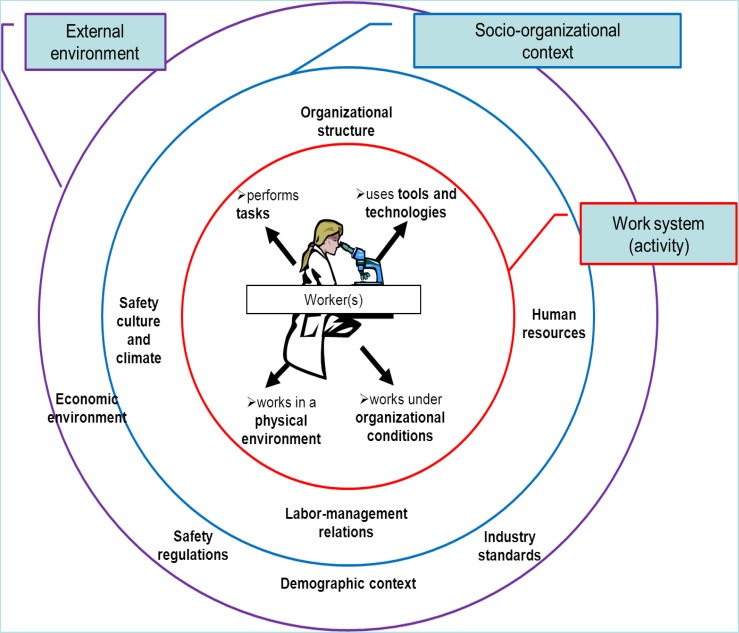
Model of sociotechnical system for workplace safety.

The STAMP model clearly outlines the relationship between system operations and system design or development. We need to complement this approach with a deeper understanding of work at the ‘sharp end’ and its relationship to the rest of the organisation and the external environment (see previous section on the role of workers in workplace safety). From a pictorial view, one can consider a sociotechnical system in the form of concentric layers, which we have illustrated in Figure 2. The proposed sociotechnical system model for workplace safety integrates the work system model of Smith and Carayon (Smith and Sainfort 1989; Carayon and Smith 2000; Carayon 2009) that represents the various elements involved in work activities; this is in line with the Francophone ergonomics tradition of ergonomic work analysis (Daniellou 2004). A larger socio-organisational context exists around the work system (activity), and includes organisational structural elements and aspects of employment relationships (Huys, Ramioul, and Van Hootegem 2011). The outer layer of the model represents the external environment and implies questions about the ultimate purpose of all such systems, and their potential for global integration (see Hancock 2009). Because the sociotechnical system is represented as a set of concentric layers, this removes the hierarchical levels that are part of the STAMP model. The elements of outer layers influence safety through both proximate and distal layers.

The innermost layer is labelled the work system and describes the *local context in which work activities are performed*. At this level, the system is viewed from a human-centric perspective and reflects the complexity of defining the multi-faceted role of the human in relation to the other elements of the system such as tasks, tools and technologies, physical environment and organisational issues (Smith and Sainfort 1989). The human is at the centre of the work system and is surrounded by a series of elements with which she/he interacts. Each system element interacts with the others; this gives rise to specific interface requirements that define a set of hardware and process specifications and criteria that address the relevant needs, capabilities and limitations of the users. Integration refers to the process of addressing human-related concerns within each system element and resolving competing demands across the entire system levels and interfaces.

The second layer, termed the social-organisational context, refers to the prevailing *social and organisational culture and structure within the enterprise*. This context moderates the efficacy of the work system design, meaning that the prevailing culture (set of core values and beliefs) and structures, influence the behaviour of humans and, in particular, the manner in which they actually interact with existing system elements (as opposed to interacting in the way envisioned by system designers). In addition to the HFE literature, the relevant management science literature on behaviour in organisational management is voluminous and often relevant here. For instance, the literature on safety culture and climate can provide the concepts and measurement techniques to examine the impact of safety culture or climate on worker behaviours in the local work system. The literature on organisational design can help to describe the organisational structures of importance to workplace safety, such as location of the safety responsibility in the organisational structure.

The outer layer represents the *social, economic, legal and political milieu or the external environment*. The broader work and occupational demographic context influences individual enterprises, their organisational culture and the specific system interfaces. This is in line with the discussion of system levels in the previous section. For instance, the STAMP model describes two levels of the external environment (see Figure 1): (1) Congress and the legislatures and (2) government regulatory agencies, industry associations, user associations, unions, insurance companies and courts. In the proposed model of sociotechnical system for workplace safety (see Figure 2), the external environment includes all of these elements, as well as the demographic context. To the extent that these influence overall performance and safety, they need to be better understood. However, a full exposition is beyond the scope of the present paper.

STS approaches, such as macro-ergonomics, describe how different system levels and their interactions can influence workplace safety. For instance, Zink (2000) showed how micro- and macro-ergonomic variables are related to each other, such as the design of tools and technologies used by workers being influenced by decisions made at the organisational level by the purchasing department. This is an example of interaction between the socio-organisational context layer and the work system layer. Training is another element of the socio-organisational context that can influence the work system layer. For instance, companies invest resources in providing safety training. The translation of that safety training to actual skills and knowledge used by the worker in the local work system is another example of the interface between the socio-organisational context and the work system. A sociotechnical systems approach to workplace safety needs to consider all the interactions among, and interfaces between, the system layers described in Figure 2.

We need to further study interactions between the layers and levels of the sociotechnical system. This research would, for example, examine how characteristics of the socio-organisational context can influence worker behaviour at the local work system level and, in turn, impact workplace safety. Future research on sociotechnical systems for workplace safety should also address the ‘active’ role of workers in ensuring safety and assess the system characteristics that promote such role. Research should also address how worker activities in the local work system can influence the socio-organisational context, such as safety culture and climate. For instance, how can an organisation provide local job control to workers so that they can manage system disturbances in a safe manner?

### 4.3. Complexity in sociotechnical systems

Principles for the design of work systems derived from STS theory can be related to complexity theory (Morin 2008). Specifically, research, design and assessment of sociotechnical systems may benefit from the growing body of insights generated from the study of complex systems within domains as diverse as computer science, biology, economics, physics and chemistry.

We suggest that sociotechnical systems are a type of *complex adaptive system*, and that analysis from that perspective could significantly enhance our understanding of how sociotechnical systems function and how they might be made to function better. Complex adaptive systems are defined as classes of complex systems whose structural and dynamic characteristics adaptively adjust in response to internal and external perturbations (Miller and Page 2007). Such systems are frequently described as ‘self-organizing’ (Pavard and Dugdale 2006) in that adaptations that occur are generally not deliberately or explicitly mandated (although, of course, this occasionally occurs in response to sufficiently disruptive events) but instead represent a quasi-organic process of redistributing activity and responsibility across the system.

Other than attention by Vicente (1999), Carayon (2006), Walker et al. (2010) and Wilson (2014), to date, there has been limited systematic exploration of the relationship between complexity theory and STS theory. As Pavard and Dugdale (2006) note, there are several attributes of complex adaptive systems that are also characteristic of complex sociotechnical systems. Among these are

- *Non-determinism*. Complex systems are fundamentally non-deterministic, as it is not possible to perfectly predict the behaviour of such systems based solely on knowledge of characteristics of its components. This is a point that has gained credence in the sociotechnical literature (Leveson 2011), and that lies at the root of the ‘Law of Unintended Consequences’, i.e. the notion that modifications to the structure or behaviour of complex sociotechnical systems commonly result in unforeseen outcomes and side effects (Pasmore 1988).
- *Limited functional decomposability.* By definition, complex systems are dynamic. The same is true of sociotechnical systems. Complexity theory asserts that attempts at analytic decomposition of such systems into constituent components cannot capture their dynamic, behavioural aspects because so much of the latter relies on interactions between system components as well as system–environment interactions.
- *Emergence and self-organisation.* Complex systems are characterised by properties and attributes that cannot be precisely localised within the structure or function of components. It is commonly noted in the sociotechnical literature that safety is an emergent property of sociotechnical system activity; yet, the factors and principles underlying the emergence of safety within such systems need to be further understood.

Because of the conceptual overlap between sociotechnical systems and the broader class of complex adaptive systems, we should further explore these connections. Within complexity theory, order and disorder are necessarily related and system evolution is largely based on this potential conflict. The goal of a system may be to impose order, but this goal is never fully achieved, not even in systems apparently in balance with their environment. One approach to complexity in sociotechnical systems is to define attributes or characteristics of system complexity (Vicente 1999; Carayon 2006; Walker et al. 2010). These complexity attributes include those noted above, as well as dynamism, uncertainty and dynamic disturbance.

One empirical question is whether sociotechnical systems are becoming more complex.^1^ There can be little doubt that the speed of their operation is increasing, as computational power increases (Moore 1965; Moravec 1990). Therefore, if one defines complexity in terms of cycle time then the issue is not in doubt. However, it may be that sociotechnical systems have always been of the same general level of interconnectivity and that rather it is our own science of HFE that has expanded its unit of analysis and, thus, now encompasses larger and more ‘complex’ work systems. Therefore, we need to develop a purpose-directed science of ‘dynamic reticulations’ or networks. We need new methods and approaches to address the ways through which dynamic patterns of interconnectedness occur and to describe the strength of interactions in the network or system. In particular, we need new methods to show how STS evolve over different timescales of action. We also need to be able to specify formal boundary conditions. While we can start with a top-down acknowledgement of the ‘system of all systems’ (Hancock 2012; Siemieniuch and Sinclair 2014), we need to distill formal rules for the inclusion or exclusion criteria of specific elements and their degree of interconnection to any specified level of the ‘system’ of interest. In essence, we, as a community, need to agree on a common definition of ‘what is a system?’ Without this agreed rubric and formalisation, the term ‘system’ and its corollary ‘sociotechnical system’ remain only generally descriptive terms that are imprecise at best and actually misleading at worst. Dynamic reticulations appear to lend themselves to modelling and simulation techniques, but there are inherent pitfalls in using representational reductions (models) in the hope of simplifying analysis when complexity itself is a central property of the phenomenon of interest. In summary, if we are to embrace the sociotechnical advances for safety improvement, we need to improve, evolve and arguably generate a revolution in our evaluative and analytic techniques.

## 5. Future challenges, aspirations and directions

When HFE was still a ‘component’ science, the idea of causational chains was appealing. Searching for causal links among a limited set of possible relationships was an important endeavour, e.g. using methods such as Markov chains. To a degree, when systems are small, finite and clearly bounded, these approaches retain their utility. Indeed, we have learned much through their use, including some of the most well-known and effective methods in HFE such as Signal Detection Theory. We should not decry nor abandon reductionist techniques to pursue STS approaches, especially when they can be helpful depending on the context of the question to answer. However, these reductionist approaches have been criticised in recent years. Innovations in non-linear dynamics have shown that even small and what previously had been conceived of as negligible influences can burgeon in non-linear ways to exert enormous and unanticipated impacts. Given that we examine ever larger and ever more interactive sociotechnical systems, we have become cognisant of the ‘emergent’ properties (e.g. safety) that such systems possess. Such emergence itself is non-linear and thus extremely hard to predict, even in engineered systems where the nominal initial conditions can be well understood. But even as we have engaged in this effort, systems themselves do not stand still. If complexity can be demarcated by the number of interacting elements, modern systems have increased their level of complexity at exponential rates. Nor can mere increased computational power alone contend with such developments since calculating faster, but on the wrong problem, actually takes us farther away, rather than nearer to, our goal of understanding.

The current state of knowledge with respect to STS theory and application to workplace safety is underdeveloped. While a myriad of models and schemas depicting interactions among system elements exist (see Table 1), several methodologies have been and continue to be developed to assist in the design and evaluation of STS performance and safety. However, many are in the conceptual stage or have been applied in a limited number of studies. Additional research is needed to enhance the scientific validation of the STS theoretical base for workplace safety. For instance, this research could compare and contrast, in a systematic manner, various levels and forms of worker participation and their impact on workplace safety. From a systems design perspective, STS approaches need to further enhance their predictive utility (Davis et al. 2014). Systems today are evolving ever more quickly and uncovering new dimensions of STS that challenge the science to catch up and keep pace.

For decades, we have developed and used physical, cognitive and psychosocial HFE methodologies to improve work systems, quality of working life and workplace safety. However, each well-meaning rule, regulation, advisory, design innovation or operational equation has met with only limited success as institutional, organisational, governmental and national barriers, beyond our apparent control or influence, have served to blunt, modulate, dissolve or simply ignore the product of our science. Sources of these constraints and barriers lie at levels of analyses beyond traditionally framed boundaries and, thus, the purview of HFE and safety professionals. With the growing interest in sociotechnical systems, we can now address these wider-scale issues. An example of this important work is that by Reason (1990), on human error, who established the relative futility of constraining our science to micro-level studies when our stated and ultimate purpose is to affect the wider system. We do not decry so-called ‘micro’-ergonomic studies, as they remain important building blocks for understanding the micro-level system design. But, apparently minor actions at one level and one sub-system can percolate across the system to negate and even reverse much stronger, clearer and rational implementations at another time and place. In order to pursue the proposed sociotechnical systems approach to workplace safety, we need more innovative and dynamic analytic methods that consider individual and momentary variation (Hancock, Hancock, and Warm 2009). For instance, we need a much more dynamic visualisation of our data fields where results can be represented by pictures in motion. Only then will a meaningful conceptualisation of sociotechnical systems in evolution be readily available to inform and support workplace safety.

## 6. Conclusion

The next significant step in improving workplace safety lies in looking beyond traditional approaches and exploring the potential of sociotechnical systems to address the fundamental challenges associated with new technologies, emerging industries and the ever-changing workforce (Dekker, Hancock, and Wilkin 2013; Holden et al. 2013; Carayon et al. 2014). This evolution will focus attention on latent or emerging risks as opposed to reacting to injuries after-the-fact. A number of relevant, yet disparate, theories and approaches can be drawn upon to understand worker safety within sociotechnical systems. Here, the focus of the theories and approaches is shifted to safety, though it is recognised that safety is an emergent property of the system and, as such, not separable from other system attributes and goals. Nevertheless, we anticipate that by deploying systems thinking to work systems, a step change can be achieved with the major public health issue of workplace safety. We argue in this respect that there is an urgent need to to develop a unified sociotechnical systems approach to workplace safety.

## Footnotes

^1.^ Note the reliance on the specific definition of ‘complex’ as derived from complexity theory. It is especially important to note that ‘complex’ does not mean the same thing as ‘complicated’.
